# The Impact of Modified Tabata Training on Segmental Fat Accumulation, Muscle Mass, Muscle Thickness, and Physical and Cardiorespiratory Fitness in Overweight and Obese Participants: A Randomized Control Trial

**DOI:** 10.3390/sports13040099

**Published:** 2025-03-26

**Authors:** Tadsawiya Padkao, Piyapong Prasertsri

**Affiliations:** Faculty of Allied Health Sciences, Burapha University, Chonburi 20131, Thailand; tadsawiya@go.buu.ac.th

**Keywords:** aerobic capacity, high-intensity intermittent training, muscle, obesity, physical performance

## Abstract

The purpose of this study was to examine changes in body fat, muscle mass, muscle thickness, and physical and cardiorespiratory fitness in overweight and obese individuals following progressive Tabata training. Thirty-six participants were randomly assigned to either the Tabata group (four progressive cycles of body-weight high-intensity intermittent training at 75–85% of maximum perceived exertion, 3 days/week for 12 weeks) or the control group. Body composition, muscle thickness, strength and endurance, and peak oxygen uptake (VO_2_peak) were measured at baseline and after the training period and compared between groups. No changes in body fat percentage and fat mass were found, but the waist-to-hip ratio was lower in the Tabata group (*p* = 0.043). The muscle mass percentages of the right (*p* = 0.026) and left legs (*p* = 0.043) increased, while the muscle thicknesses of the biceps, triceps, rectus femoris, and vastus intermedius were increased in the Tabata group (*p* < 0.05) to a greater extent than in the control group (*p* < 0.05). Muscle strength and endurance (*p* < 0.05), as well as VO_2_peak (*p* = 0.006), also increased in the Tabata group. Twelve weeks of modified Tabata training effectively increased muscle mass and thickness and physical and cardiorespiratory fitness, although it did not reduce fat mass in overweight and obese participants. The combination of this training with a dietary intervention may have a more obvious impact.

## 1. Introduction

Obesity is a major global health concern [1], with the prevalence of overweight and obesity individuals having doubled worldwide; in 2022, 16% of adults were overweight and 43% were obese [2]. These conditions present major risks for cardiovascular, neurodegenerative, respiratory, autoimmune, non-communicable, and musculoskeletal diseases, in addition to major cancers [3,4,5].

Typically, the management of overweight and obesity involves weight control, reducing abdominal fat, and improving cardiorespiratory fitness, with approaches to these goals including dietary therapy, exercise, lifestyle changes, medication, and bariatric surgery [6]. The American College of Sports Medicine [7] recommends moderate-to-vigorous-intensity aerobic exercise as a method of weight control.

High-intensity intermittent training (HIIT) is a form of interval training that alternates brief periods of intense exercise with complete rest [8]. The duration of the exercise and rest periods varies from 6 s to 4 min over a period of 2 to 15 weeks [9]. HIIT activities such as sprinting or cycling have been shown to reduce body fat, abdominal fat, and waist circumference with minimal weight loss, while improving maximal oxygen consumption (VO_2_max) in obese children, healthy adolescents, overweight women, and adults with type 2 diabetes [10,11]. Additionally, an 8-week Tabata HIIT program involving sedentary men increased their levels of irisin, a hormone that aids in fat utilization [12].

Tabata training, a form of HIIT, involves 20 s of intense exercise followed by 10 s of rest, repeated for 7–8 sets [13]. Originally developed for cycling [14], it has been adapted for other forms of exercise like running and body-weight workouts. The benefits of Tabata training include burning fat, heightened metabolism during and after workouts, and the enhancement of anaerobic and aerobic systems [13,14,15]. Previous studies have shown that 4 to 12 weeks of body-weight Tabata training can increase VO_2_max by 5–18% [16].

Most studies on the Tabata protocol have been conducted on healthy men and women or (primarily) athletes with low physical fitness levels, where improvements in muscle gene expression related to both sports performance and health promotion have been demonstrated [16,17]. Given the high intensity of Tabata training, adherence may be challenging; for example, a study of obese male adolescents reported a 6.3% withdrawal rate [18] and 90% compliance in sedentary participants [19]. These findings suggest that this intensity may reduce enjoyment for some participants [20], and a lower-intensity regimen could enhance motivation. The body-weight exercises commonly used in Tabata training, such as squats, jumps, and lunges, involve high-impact movements which may be unsuitable for overweight individuals, highlighting the need for modifications.

We conducted a literature review and identified the following research gaps: (1) HIIT with the Tabata protocol appears to enhance fat utilization by increasing serum irisin levels, which could benefit individuals seeking to reduce body fat; (2) the very high intensity of HIIT may reduce exercise compliance due to decreased enjoyment, particularly in adolescents and young adults; (3) high-impact exercises may not be suitable for overweight or obese individuals; and (4) the effectiveness of Tabata protocols in promoting weight loss has been examined in only a few previous studies [16].

As such, in this study, we aimed to examine whether modified Tabata HIIT changes body fat, fat mass, muscle mass, muscle thickness, and physical and cardiorespiratory fitness. The hypothesis was that the modified Tabata training would improve on these variables in overweight and obesity participants.

## 2. Materials and Methods

### 2.1. Study Design: Randomized and Non-Blinded

**Figure 1:**
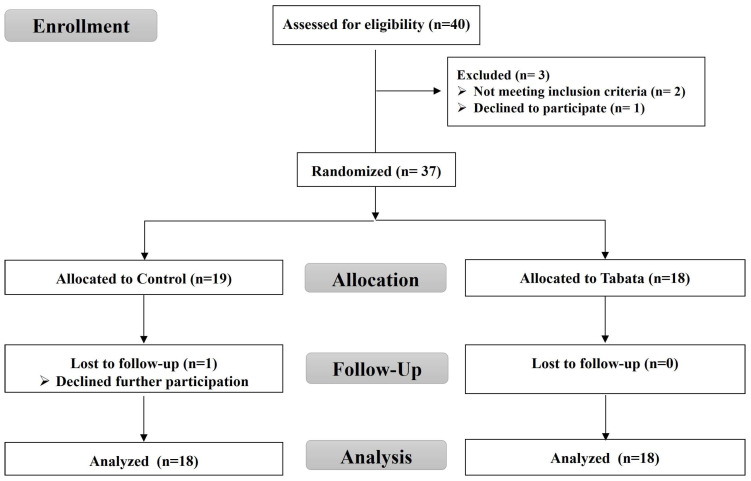
CONSORT flow diagram.

For this randomized controlled trial, as shown in the CONSORT flow diagram (Figure 1), we recruited overweight or obese individuals living in Chonburi Province starting in September 2022. This study used a stratified blocked randomization method to assign subjects to either the control or Tabata group using the RAND function in Microsoft Excel. This study was non-blinded, with the same researcher responsible for screening, randomization, allocation, data collection, and analysis.

This study was approved by the Burapha University Institutional Review Board on 24 May 2022 (ID: G-HS018/2565) and registered in the Thai Clinical Trials Registry (ID: TCTR20220518001). All participants gave written informed consent prior to screening.

### 2.2. Screening of Participants

Participants aged 18–30 years with a BMI > 22.9 kg/m^2^ [21] were included. The exclusion criteria were the consumption of certain drugs; cardiovascular, liver, renal, musculoskeletal, infectious, neurological, or psychiatric diseases or cancer; and regular smoking and alcohol consumption. A health history questionnaire was designed based on the inclusion and exclusion criteria and was used to screen for eligibility. It included questions about personal information such as gender, age, smoking history, use of supplements or tonics, history of lithium use, pregnancy or breastfeeding history, and various diseases specified in the inclusion and exclusion criteria. Additionally, the Baecke Habitual Physical Activity Questionnaire was used to determine activity levels (sedentary or active) [22,23]. A research assistant administered the health history and activity level questionnaires to the volunteers. These questionnaires were completed before the participants were divided into groups.

### 2.3. Sample Size

The sample size was calculated using the means and standard deviations of VO_2_max according to a previous study [24] that investigated the effects of a 4-week Tabata HIIT intervention in overweight individuals. With an α error of 0.05 and a test power of 0.98, the sample size was obtained using G*Power 3.1 [25]; accounting for a 10% dropout, the sample size was 18 per group (36 total).

### 2.4. Tabata Training

**Figure 2:**
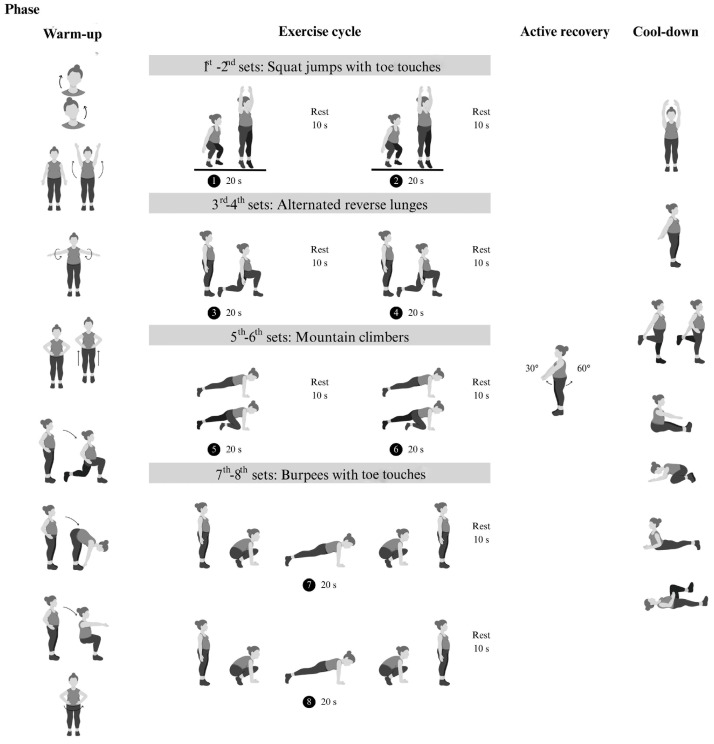
Phases of exercise training: warm-up, 4 min exercise cycle (body-weight-bearing based on Tabata training method), 4 min active recovery, and cool-down.

The modified Tabata training was a progressive home-based program consisting of body-weight exercises followed by active rest (Figure 2). This progressive body-weight HIIT program was designed based on biomechanical principles. Four body-weight exercises, including squat jumps with toe touches and alternating reverse lunge movements, primarily targeted the leg muscles (quadriceps, gastrocnemius) and core stabilizers (transversus abdominis, multifidus). Additionally, mountain climbers and burpees with toe touches movements engaged the upper limb muscles (biceps, triceps, pectoralis) in addition to the leg and core muscles. This training was designed to reduce exhaustion and prevent dropout [20]. To minimize the impact on the lower limb joints and reduce the risk of injury during unsupervised exercise, we modified the squat jumps and burpees by replacing jumps with standing on tiptoes. Each session included 4 min exercise cycles (20 s of exercise and 10 s of rest) followed by 4 min of active rest, totaling 8 min per cycle. Participants trained three days a week for 12 weeks, starting with two cycles for the first 4 weeks, then increasing to three cycles from weeks 5 to 8 and four cycles from weeks 9 to 12. The exercise intensity was set at 75–85% of maximum perceived exertion, with 4 min active rest periods where participants swung their arms at 40–50% exertion. Participants were provided with an exercise diagram and video clip to demonstrate the exercises. The exercise area was approximately 3 m in width and 3 m in length. The researcher monitored the correctness of exercise posture and compliance with the exercise plan. Appointments were scheduled for measurement and exercise sessions for each volunteer, and communication was conducted via online conference platforms such as Line, Google Meet, or Microsoft Teams, depending on convenience. During the session, the exercise video clip was played, and the researcher observed and provided real-time feedback to correct posture as needed while offering encouragement throughout the exercises.

Exercise intensity was assessed using the rating of perceived exertion (RPE) scale developed by Gunnar Borg [26]. This scale is a numerical rating system ranging from 6 (no exertion) to 20 (maximum effort). A rating of 7 to 11 represents extremely light to light intensity, 12 to 14 indicates moderate to somewhat hard intensity, and 15 to 17 represents hard to very hard intensity. Prior to data collection, the meaning of each rating was explained to the participants during the pre-test day. They were instructed to assess their exertion level during each set of exercises performed at home. Researchers recorded individual RPE scores to monitor exercise intensity and provided real-time encouragement to ensure participants reached the target intensity range specified in the exercise program.

The control group participants were asked to maintain their current physical activity and diet.

### 2.5. Study End Points

The primary outcomes were improvements in body composition, including body fat, fat mass, muscle mass, and muscle thickness. The secondary outcomes were improvements in fat distribution, including waist and hip circumference and ratio; physical fitness, including muscle strength and endurance; and cardiorespiratory fitness, including aerobic capacity. Both outcomes were measured before and after the 12-week interventions.

All outcome measurements were conducted by the same researcher, a certified physical therapist trained by an exercise biology expert before the study commenced. The measurements took place between 08:00 and 12:00 on the appointment date at the Exercise and Nutrition Innovation and Sciences Research Unit Room, Faculty of Allied Health Sciences, Burapha University. The order of measurements on each day is provided below.

#### 2.5.1. Body Composition and Vital Signs

Body composition, including body weight, BMI, fat percentage, fat and muscle mass, water, protein, mineral, visceral fat, and basal metabolic rate, was measured using a bioelectrical impedance analyzer (InBody270, InBody Co., Ltd., Daejeon, Republic of Korea) [27].

Resting vital signs, including heart rate (HR), respiratory rate (RR), systolic and diastolic blood pressure (SBP and DBP), mean arterial pressure (MAP), and partial oxygen saturation (SpO_2_), were measured using a real-time vital sign bedside monitor (PVM-2701 Vismo, Nihon-Kohden, Tokyo, Japan) after the participant had been seated for 10 min. Each measurement was taken twice, with a 2 min interval between readings, and the average of the two values was reported.

#### 2.5.2. Muscle Thickness

An ultrasound machine (M5 series, Shenzhen Mindray Bio-Medical, Shenzhen, China) with a 7.5 MHz probe was used to measure two upper limb muscles (biceps and triceps brachii) and two lower limb muscles (rectus femoris and vastus intermedius) in the dominant limb according to a published protocol [28,29]. Measurements were taken twice and analyzed using ImageJ software version 1.54g (Wayne Rasband, NIH, Bethesda, MD, USA), which showed a high intraclass correlation coefficient (0.999 for biceps and triceps, 0.996 for rectus femoris, and 0.989 for vastus intermedius). The average thickness was calculated from two images for analysis.

#### 2.5.3. Physical Fitness

Lower-body muscle strength was assessed using the Leg Dynamometer Test [30] (Takei Back/Leg Dynamometer Digital, Tokyo, Japan), measuring quadriceps strength in kilograms. Upper-body muscle endurance was evaluated with the YMCA Bench Press Test [30], recording total successful repetitions (reliability r = 0.87).

#### 2.5.4. Cardiorespiratory Fitness

An incremental treadmill test (Valiant 2 Sport, Lode, Groningen, The Netherlands) following the Bruce protocol [31] was used to determine the peak VO_2_ (VO_2_peak). Gas and volume calibrations were carried out before each test following the manufacturer’s guidelines. Breath-by-breath analysis was measured with a cardiopulmonary exercise testing system and software (MetaMax 3B, Cortex, Leipzig, Germany). HR, breathing frequency (BF), VO_2_, carbon dioxide production (VCO_2_), respiratory exchange ratio (RER), first ventilatory threshold (VT_1_), and second ventilatory threshold (VT_2_) were measured in real time and analyzed. The test was terminated when the participant reached 75–95% of maximum HR (HRmax), expressed volitional fatigue, or met the guideline termination criteria [7].

### 2.6. Statistical Analysis

Data were analyzed using SPSS version 26 (IBM Corp., Armonk, NY, USA) and expressed as mean ± standard deviation or median (range). The Kolmogorov–Smirnov test assessed normality, after which appropriate parametric or non-parametric tests were applied. Differences between groups were analyzed using the independent *t*-test. Since the body composition data were not normally distributed, non-parametric tests (e.g., the Mann–Whitney U test) were employed. Pre- and post-test differences within each group were analyzed utilizing the dependent *t*-test, except for the bench press test, which used the Wilcoxon signed-rank test. Statistical significance was set at *p* < 0.05. The effect size (*ES*) was calculated following Cohen’s guidelines, with values above 0.8 denoting large effects, 0.5–0.8 medium effects, and 0.2–0.5 small effects.

## 3. Results

### 3.1. Participant Characteristics and Feasibility

A total of 40 volunteers initially registered, with 37 meeting the criteria, 36 completing the study, and 1 control group participant withdrawing. The study enrollment process is illustrated in Figure 1. The baseline physical and physiological characteristics, as well as vital signs, are detailed in Table 1, with no significant differences between groups (all *p* > 0.05).

None of the Tabata group dropped out, and no serious adverse events occurred. The RPE ranged from 7 to 11 during the warm-up period, 13 to 17 during exercise, and 9 to 11 during the cool-down period. Minor adverse events, including leg muscle cramps and dizziness in two participants, occurred during the fourth cycle of the final week, representing 11.11% of the training group. However, no participants discontinued the training. No participants experienced joint injuries, thus confirming our initial presuppositions.

### 3.2. Impact on Body Composition

The pretest body composition showed no significant differences between groups (Table 2, Table 3 and Table 4). After 12 weeks of training, changes in segmental muscle composition were observed. In the control group, the muscle mass and muscle percentage of the right leg, in addition to the muscle percentage of the left leg, decreased compared to the pre-test values (all *p* < 0.05). The Tabata group showed no significant change in muscle mass in both legs. Post-test comparisons between groups revealed that the Tabata group had a higher right leg muscle mass (0.18 ± 0.08 kg (*p* = 0.031, *ES* = 0.22)) and a higher muscle percentage in both legs (right: 2.08 ± 0.89%, *p* = 0.026, *ES* = 0.63; left: 1.82 ± 0.86%, *p* = 0.043, *ES* = 0.67). The control group showed increases in abdominal fat and fat percentage (*p* < 0.05), while the Tabata group showed no changes. Notably, the Tabata group had a significantly lower waist-to-hip ratio (WHR) (0.02 ± 0.01) relative to the control group (*p* = 0.043, *ES* = 0.83).

### 3.3. Impact on Muscle Thickness

**Figure 3:**
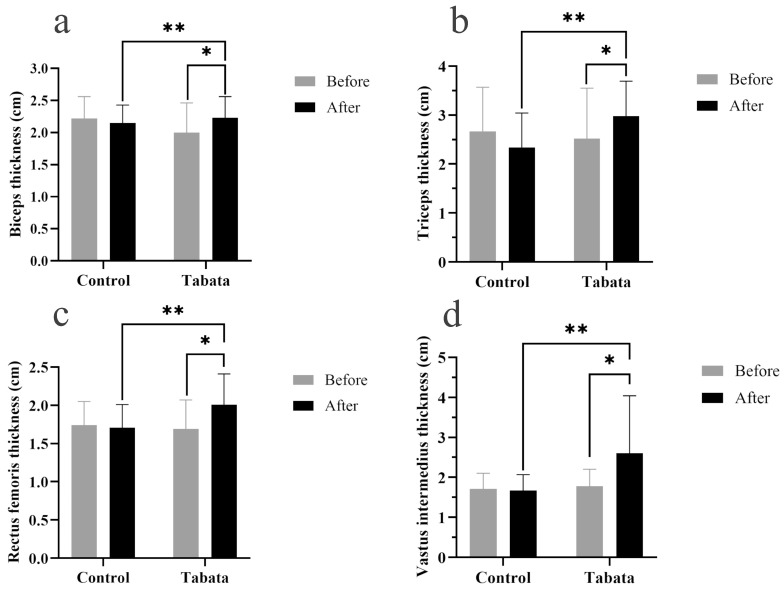
Biceps muscle thickness (**a**), triceps muscle thickness (**b**), rectus femoris muscle thickness (**c**), and vastus intermedius muscle thickness (**d**) of participants in control and Tabata groups before and after 12-week intervention. *: *p* < 0.05 vs. before intervention, **: *p* < 0.05 vs. control group.

**Figure 4:**
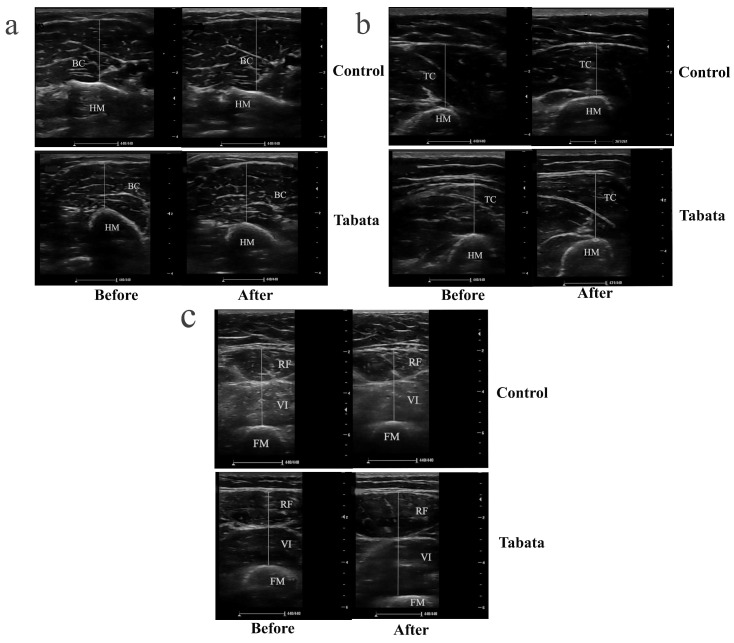
Ultrasound images demonstrating muscle thickness of participants in control and Tabata groups before and after 12-week intervention. (**a**) Transverse scan of biceps muscle (BB), with the hyperechoic, curvilinear structure in the lower part of the image representing the humerus (HM). (**b**) Transverse scan of triceps muscle (TC), where the hyperechoic, curvilinear structure in the lower part of the image corresponds to the HM. (**c**) Transverse scan of quadriceps muscle, comprising rectus femoris (RF) and vastus intermedius (VI); the hyperechoic, curvilinear structure in the lower part of the image represents the femur (FM). Solid straight lines indicate the method used to measure the thickness of each muscle.

The Tabata group showed significant increases in muscle thicknesses of 0.30 ± 0.08 cm (biceps), 0.78 ± 0.25 cm (triceps), 0.35 ± 0.10 cm (rectus femoris), and 0.85 ± 0.34 cm (vastus intermedius) compared to both the pre-test period (all *p* < 0.05) and the control group (*p* = 0.001, *ES* = 0.26; *p* = 0.028, *ES* = 0.90; *p* = 0.003, *ES* = 0.84; *p* = 0.004, *ES* = 0.88, respectively), as shown in Figure 3 and Figure 4.

### 3.4. Impact on Physical Fitness

**Figure 5:**
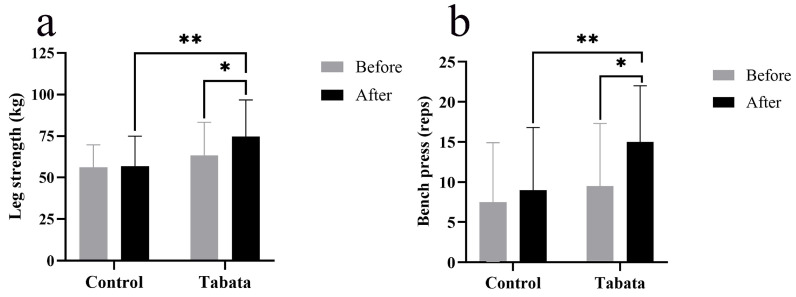
Leg muscle strength (**a**) and arm muscle endurance (bench press) (**b**) of participants in control and Tabata groups before and after 12-week intervention. *: *p* < 0.05 vs. before intervention, **: *p* < 0.05 vs. control group.

The Tabata group showed improvements in lower-body muscle strength, as evidenced by a 10.93 ± 4.27 kg increase in weight lifted compared to both the pre-test period (*p* = 0.004) and the control group (*p* = 0.015, *ES* = 0.89) (Figure 5a). Upper-body muscle endurance also improved, as indicated by a 5.00 ± 7.80 increase in bench press repetitions compared to both the pre-test period (*p* < 0.001) and the control group (*p* < 0.001, *ES* = 0.47) (Figure 5b).

### 3.5. Impact on Cardiorespiratory Fitness

**Figure 6:**
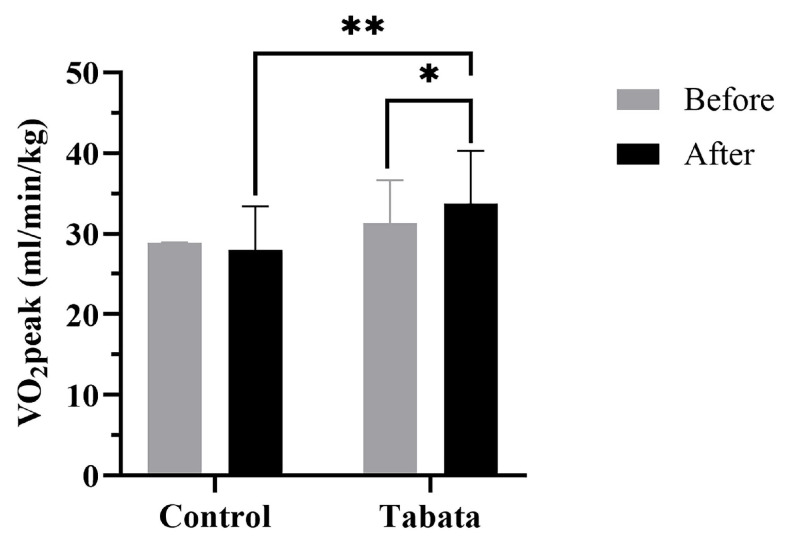
Peak oxygen consumption (VO_2_peak) of participants in control and Tabata groups before and after 12-week intervention. *: *p* < 0.05 vs. before intervention, **: *p* < 0.05 vs. control group.

There were no significant differences in cardiorespiratory fitness shown between the two groups in the pre-test period (Table 5 and Figure 6). After 12 weeks, the control group showed a decrease in resting VO_2_ of 0.50 ± 0.70 mL/min/kg (*p* = 0.008). The Tabata group demonstrated greater improvements in anaerobic threshold and aerobic capacity, with increased VT_2_ (2.33 ± 1.53 mL/min/kg; *p* = 0.013) and VO_2_peak (3.33 ± 1.12 mL/min/kg; *p* = 0.006, *ES* = 0.56). Breathing frequency and RER increased in both groups, but no significant differences were seen between groups.

## 4. Discussion

This randomized control study provides evidence that the 12 weeks of modified Tabata training was safe and effective for overweight and obese participants. The program reduced WHR, enhanced leg muscle mass, and increased arm and leg muscle thickness, which collectively resulted in improvements in leg strength and arm endurance as well as aerobic capacity.

### 4.1. Modified Tabata Training Improves Body Proportions

A recent systematic review revealed that HIIT has the potential to facilitate weight loss in overweight and obese individuals [32]. Other investigations also reported positive effects, such as a 12-week HIIT program reducing body weight by 5.7 kg (8.3–3.1 kg) in obese adults [33,34]. However, the Tabata group did not demonstrate changes in either body weight or BMI; only minor changes were observed, similar to a previous study [35]. Furthermore, a systematic review also showed that HIIT can induce modest body composition improvements in overweight individuals without affecting body weight [36].

Following the 12-week HIIT intervention, the Tabata group demonstrated reduced WHR, leading to a more proportionate physique, similar to a previous study that showed reduced WHR and visceral fat in obese women [37]. However, only minimal reductions in other body composition indices were observed, which might be attributed to the lower intensity of exercise in this study (75–85% HRmax) relative to previous studies which reported more positive results (90–95% HRmax). This highlights the fact that heterogenous intensity and duration result in diverse outcomes. For example, the 12-week HIIT protocol reported by Astorino et al. (~75–95% HRmax) did not affect body weight or WHR [38]. Similarly, two recent studies revealed no changes in body weight or body composition in sedentary, overweight, or obese individuals following HIIT [39]. From a mechanistic point of view, two possible explanations for this lack of change are (1) behavioral adaptations that lead to an increase in food intake immediately following exercise, such as the consumption of snacks high in carbohydrates and fats, and (2) metabolic adaptations associated with the addition of a high-intensity exercise program. Specifically, a reduction in non-exercise activity thermogenesis (NEAT) may have occurred as a compensatory response to the increased exercise-induced energy expenditure in the experimental group [40,41].

### 4.2. Modified Tabata Training Improves Muscle Thickness

HIIT has been demonstrated to increase muscle thickness effectively in overweight and obese populations. Previous studies reported increases in vastus lateralis muscle thickness after 12 weeks of HIIT (Δ change = −3.17 ± 3.36 cm^2^) compared to traditional strength training, e.g., sprints (Δ change = −0.34 ± 2.36 cm^2^). The effects of HIIT on upper-body muscle thickness are less well documented; however, when HIIT protocols incorporate upper-body resistance exercises, such as push-ups, they also lead to increased muscle thickness [42]. Meanwhile, this study’s findings align with previous studies in which exercise types engaging both the upper and lower limb muscles, e.g., mountain climbers and burpees with toe touches, led to increased upper limb muscle thickness [43].

The hypertrophic effects of HIIT are attributed to increased muscle fiber recruitment, metabolic stress, and hormonal responses, including elevated levels of growth hormone, testosterone, and estrogen, all of which are linked to muscle growth [44,45,46]. The nature of HIIT induces metabolic stress, which is a key factor driving muscle hypertrophy [47].

### 4.3. Modified Tabata Training Improves Muscle Strength and Endurance

Studies have demonstrated that HIIT, particularly when involving sprinting or jumping, improves lower-body strength due to its high intensity [48]. Again, the effects on upper-body strength are generally less pronounced; however, HIIT protocols including upper-body resistance exercises such as push-ups can lead to improvements in this regard [49]. Our study is consistent with prior research, in that exercises involving both upper and lower limb muscles were shown to contribute to increased muscle strength [40]. The enhancement of strength caused by HIIT is thought to be a result of several factors, including the recruitment of fast-twitch muscle fibers and neuromuscular adaptations [50].

In addition, studies on HIIT’s effects on muscle endurance are limited, though HIIT has been demonstrated to augment muscle endurance by enhancing muscular enzyme content and activity [15]. A 4-week running and body-weight HIIT study reported enhanced muscle endurance, as evidenced by more burpees and toe-to-bar repetitions being performed [51]. These findings are consistent with the increase in bench press repetitions in our study. Moreover, sprint and cycling HIIT increased mitochondrial [52] and glycolytic enzyme activities [17]. These changes indicate that HIIT enhances protein expression, muscle adaptation, and energy expenditure in both anaerobic and aerobic systems.

### 4.4. Modified Tabata Training Improves Cardiorespiratory Fitness

The Tabata group demonstrated enhanced aerobic capacity. In line with this, a systematic review of 15 randomized controlled trials indicated that HIIT was more effective than traditional exercise in increasing VO_2_max [35]. Previous studies with obese and elderly participants also showed that 12–16 weeks of HIIT effectively improved VO_2_max and cardiovascular health [53,54]. A potential change after modified Tabata HIIT is enhanced muscle buffer capacity, which results in proportional glycolytic ATP production [55,56]. Furthermore, as reported in a review by Torma et al. [57], HIIT activates key pathways that enhance mitochondrial biogenesis and angiogenesis in skeletal muscle.

After identifying significant differences between the control and Tabata groups, Pearson correlation analysis revealed a positive correlation between VO_2_peak and VT_2_ (*r* = 0.844, *p* < 0.001), while Kendall correlation analysis identified a negative relationship between WHR and VT_2_ (*r* = −0.456, *p* = 0.008). The observed relationships suggest that modified Tabata training may enhance VO_2_peak, potentially due to the more efficient utilization of anaerobic energy pathways. Furthermore, as the proportion of visceral fat increases, aerobic energy pathway efficiency decreases.

Despite variations in exercise protocols, such as cycling, running in place, or calisthenics, most studies have found little to no difference in the effects of HIIT, provided that the exercise duration and rest periods range from 6 s to 4 min and the intensity reaches 75% of HR or VO_2_max [9]. A previous study comparing two HIIT protocols in 11 college students aged 19–27 years examined sprint interval cycling (four 30 s sets with a 4 min active recovery) versus high-intensity intermittent calisthenics (four sets of ten squats, ten push-ups, and five burpees, each performed for 30 s with a 4 min active recovery) over 9 days. The results showed no statistically significant differences between the two protocols in terms of %VO_2_peak (80.4 ± 5.3% vs. 77.6 ± 6.9%, respectively) [58]. Similarly, a 9-week study investigating the effects of four different high-intensity training modalities (HIIT, high-intensity functional training, high-intensity power training, and high-intensity endurance training) found that all four groups experienced an 8% improvement in VO_2_max, while the levels of brain-derived neurotrophic factor (BDNF), a key regulator of neurogenesis, remained unchanged in all groups [59]. These findings suggest that regardless of the specific exercise modality, when exercise and rest durations range from 6 s to 4 min over a period of 2 to 15 weeks, similar physiological benefits can be achieved [9].

### 4.5. Limitations of Study

This study has several limitations. Firstly, the exercise program for the Tabata group was home-based, with an adherence rate of 89.87% and a compliance rate of 100%. Exercise frequency and intensity were monitored online, and participants recorded their perceived exertion. To ensure accuracy and maintain intensity, participants were required to visit the research room every 4 weeks for guidance amid subsequent sessions. Although monitoring took place online, supervised programs may yield better adherence and control. Future studies should implement supervised exercise programs in a controlled setting and HR tracking should be used to enhance reliability. Secondly, eating behaviors were not strictly controlled due to being ecologically practicable and reliant on self-reported data. This limitation is particularly relevant to the control group, as changes in body composition could have been influenced by unreported dietary factors. In future research, implementing more stringent dietary control or more reliable monitoring methods is recommended. Thirdly, the impact of variables like increased protein intake and metabolic hormones (e.g., growth hormone, testosterone) was unaccounted for, and should thus be considered by future studies. Lastly, body composition data were obtained using an electrical impedance technique. While this method is highly reliable, more accurate techniques, such as Dual-Energy X-ray Absorptiometry (DEXA) or the BodPod, should be considered for directly measuring body composition, if feasible.

## 5. Conclusions

Modified Tabata training, a progressive body-weight HIIT program taking place over 12 weeks, proved safe and effective for overweight and obese participants. Improvements in WHR, leg muscle mass, and arm and leg muscle thickness were seen; furthermore, these improvements led to enhancements in physical fitness due to increased muscle strength and endurance, in addition to enhancements in cardiorespiratory fitness through increased aerobic capacity. These results suggest that this training method has potential in terms of improving body proportion and overall health among overweight and obese populations.

## Figures and Tables

**Table 1:** Physical and physiological characteristics and vital signs of participants.

	Control Group (*n* = 18)	Tabata Group (*n* = 18)
Number	18	18
Sex (n, male:female)	5:13	5:13
Age (years)	21.61 ± 2.06	20.72 ± 1.32
WHO BMI classification (n, overweight:obese)	2:16	5:13
Physical activity score		
- Pre-test	7.01 ± 1.07	7.04 ± 0.99
- Post-test	6.96 ± 0.79	7.31 ± 1.22
Physical activity level		
- Sedentary (n, %)	3 (17%)	3 (17%)
- Active (n, %)	11 (61%)	12 (66%)
- Athletic (n, %)	4 (22%)	3 (17%)
Resting HR (/min)	77.22 ± 9.35	77.00 ± 9.83
Resting RR (/min)	18.33 ± 3.29	16.11 ± 2.03
Resting SBP (mmHg)	118.65 ± 16.16	118.31 ± 11.77
Resting DBP (mmHg)	74.41 ± 12.54	73.94 ± 8.54
Resting MAP (mmHg)	89.15 ± 13.04	88.73 ± 9.00
Resting SpO_2_ (%)	97.78 ± 1.21	97.50 ± 1.29

BMI, body mass index; DBP, diastolic blood pressure; HR, heart rate; MAP, mean arterial pressure; RR, respiratory rate; SBP, systolic blood pressure; SpO_2_, partial oxygen saturation; WHO, World Health Organization.

**Table 2:** Whole-body composition of participants.

	Control Group (*n* = 18)			Tabata Group (*n* = 18)			*p*-Value of Change Between Groups
	Pre-Test	Post-Test	Mean Change	Pre-Test	Post-Test	Mean Change	
BW (kg)	74.25 ± 19.70	73.85 ± 17.80	−0.20 ± 4.77	73.85 ± 28.05	74.70 ± 25.80	1.45 ± 2.78	0.281
Height (cm)	163.00 ± 9.06	163.11 ± 8.86	0.00 ± 0.00	163.00 ± 9.06	163.16 ± 8.77	−0.05 ± 0.23	0.956
BMI (kg/m^2^)	27.10 ± 5.15	28.00 ± 4.72	−0.05 ± 1.73	27.00 ± 7.47	27.75 ± 7.98	0.40 ± 1.13	0.269
MM (kg)	23.35 ± 7.80	23.55 ± 7.78	−0.30 ± 1.15	23.30 ± 9.85	23.60 ± 10.55	0.20 ± 1.38	0.290
FMs (kg)	31.00 ± 11.13	30.00 ± 9.52	0.25 ± 2.68	29.20 ± 15.20	30.85 ± 14.48	0.75 ± 3.97	0.529
BF (%)	40.20 ± 10.25	39.60 ± 10.73	0.80 ± 2.22	41.10 ± 11.90	39.90 ± 15.02	−0.15 ± 3.90	0.927
FFM (kg)	42.65 ± 13.02	42.80 ± 12.93	−0.70 ± 1.88	42.75 ± 16.63	43.15 ± 17.00	0.45 ± 2.48	0.361
BMR (kcal)	1291.50 ± 281.25	1295.00 ± 280.25	−16.00 ± 41.25	1293.50 ± 359.75	1302.00 ± 368.00	9.5 ± 53.75	0.361
Water (L)	31.20 ± 9.55	31.30 ± 9.67	−0.45 ± 1.33	31.30 ± 12.08	31.60 ± 12.50	0.25 ± 1.83	0.384
Protein (kg)	8.45 ± 2.68	8.50 ± 2.55	−0.10 ± 0.35	8.40 ± 3.20	8.50 ± 3.60	0.10 ± 0.33	0.300
Mineral (kg)	3.11 ± 0.87	3.01 ± 0.80	−0.05 ± 0.14	2.98 ± 1.11	3.00 ± 1.10	0.10 ± 0.23	0.293
WHR	0.92 ± 0.06	0.94 ± 0.06 *	0.02 ± 0.03	0.91 ± 0.06	0.89 ± 0.06 **	−0.01 ± 0.03	0.043

BF, body fat; BMI, body mass index; BMR, basal metabolic rate; BW, body weight; FFM, fat-free mass; FMs, fat mass; MM, muscle mass; WHR, waist–hip ratio. *: *p* < 0.05 vs. before intervention, **: *p* < 0.05 vs. control group.

**Table 3:** Segmental muscular composition of participants.

	Control Group (*n* = 18)			Tabata Group (*n* = 18)			*p*-Value of Change Between Groups
	Pre-Test	Post-Test	Mean Change	Pre-Test	Post-Test	Mean Change	
Right arm (kg)	2.38 ± 0.66	2.40 ± 0.65	0.01 ± 0.10	2.44 ± 0.70	2.45 ± 0.71	0.01 ± 0.10	0.937
Right arm (%)	93.38 ± 9.00	94.08 ± 8.17	0.70 ± 4.36	96.60 ± 12.35	96.80 ± 13.30	0.20 ± 3.51	0.707
Left arm (kg)	2.35 ± 0.64	2.35 ± 0.63	−0.00 ± 0.11	2.38 ± 0.66	2.43 ± 0.69	0.04 ± 0.10	0.198
Left arm (%)	92.20 ± 8.17	92.11 ± 7.05	−0.09 ± 4.76	94.78 ± 12.71	95.98 ± 13.50	1.19 ± 3.51	0.363
Right leg (kg)	7.20 ± 1.50	7.04 ± 1.49 *	−0.16 ± 0.24	7.41 ± 1.88	7.43 ± 1.88 **	0.02 ± 0.24	0.031
Right leg (%)	93.71 ± 6.55	91.56 ± 6.11 *	−1.72 ± 2.58	96.18 ± 8.03	96.22 ± 8.44 **	0.06 ± 2.45	0.026
Left leg (kg)	7.13 ± 1.48	6.84 ± 1.78	−0.29 ± 0.84	7.38 ± 1.83	7.42 ± 1.84	0.04 ± 0.25	0.117
Left leg (%)	92.78 ± 6.02	91.06 ± 6.26 *	−1.72 ± 2.58	95.87 ± 7.59	95.97 ± 8.23 **	0.10 ± 2.60	0.043
Abdomen (kg)	20.75 ± 4.10	20.79 ± 4.06	0.04 ± 0.61	20.95 ± 4.30	21.17 ± 4.37	0.22 ± 0.52	0.358
Abdomen (%)	94.56 ± 5.16	94.80 ± 4.49	0.23 ± 2.80	95.99 ± 7.25	96.50 ± 7.84	0.50 ± 2.11	0.750

*: *p* < 0.05 vs. before intervention, **: *p* < 0.05 vs. control group.

**Table 4:** Segmental fat composition of participants.

	Control Group (*n* = 18)			Tabata Group (*n* = 18)			*p*-Value of Change Between Groups
	Pre-Test	Post-Test	Mean Change	Pre-Test	Post-Test	Mean Change	
Right arm (kg)	2.69 ± 1.38	2.63 ± 1.32	0.33 ± 0.21	2.58 ± 1.50	2.71 ± 1.56	0.09 ± 0.25	0.445
Right arm (%)	321.72 ± 161.12	324.39 ± 153.33	2.67 ± 25.11	308.11 ± 170.64	322.76 ± 43.70	11.03 ± 31.57	0.385
Left arm (kg)	2.62 ± 1.37	2.66 ± 1.32	0.04 ± 0.22	2.63 ± 1.50	2.67 ± 1.54	0.07 ± 0.25	0.675
Left arm (%)	324.87 ± 161.29	329.37 ± 154.56	4.50 ± 25.21	313.80 ± 173.20	319.14 ± 183.72	8.95 ± 31.53	0.643
Right leg (kg)	4.85 ± 1.93	4.72 ± 1.59	−0.12 ± 0.56	4.75 ± 1.74	4.87 ± 1.94	0.12 ± 0.41	0.138
Right leg (%)	226.24 ± 82.10	220.52 ± 69.46	−5.71 ± 23.97	217.58 ± 80.25	222.93 ± 89.87	5.29 ± 18.89	0.134
Left leg (kg)	4.82 ± 1.89	4.68 ± 1.58	−0.13 ± 0.52	4.72 ± 1.71	4.85 ± 1.90	0.12 ± 0.39	0.100
Left leg (%)	225.03 ± 80.70	219.66 ± 68.57	−5.36 ± 23.46	216.13 ± 78.25	221.43 ± 88.22	5.35 ± 18.93	0.142
Abdomen (kg)	15.20 ± 3.84	15.58 ± 3.88 *	0.37 ± 0.82	15.05 ± 4.83	15.26 ± 1.18	0.21 ± 1.11	0.625
Abdomen (%)	316.30 ± 88.06	324.00 ± 87.19 *	7.70 ± 16.94	305.77 ± 102.02	309.73 ± 108.02	3.96 ± 23.14	0.583

*: *p* < 0.05 vs. before intervention.

**Table 5:** Cardiorespiratory fitness of participants.

		Control Group (*n* = 18)			Tabata Group (*n* = 18)			*p*-Value of Change Between Groups
		Pre-Test	Post-Test	Mean Change	Pre-Test	Post-Test	Mean Change	
Rest								
	HR (beats/min)	94.44 ± 10.31	92.00 ± 11.90	−2.44 ± 7.51	91.72 ± 10.39	90.16 ± 10.40	−1.55 ± 8.84	0.747
	BF (breaths/min)	20.16 ± 3.85	19.50 ± 3.83	−0.66 ± 2.97	21.05 ± 4.24	21.66 ± 5.20	0.61 ± 2.70	0.186
	RER	0.80 ± 0.05	0.84 ± 0.03 *	0.04 ± 0.06	0.81 ± 0.04	0.86 ± 0.04 *	0.04 ± 0.06	0.740
	VO_2_ (L/min)	0.33 ± 0.07	0.29 ± 0.07 *	−0.04 ± 0.04	0.36 ± 0.07	0.34 ± 0.08 **	−0.02 ± 0.06	0.265
	VO_2_ (ml/min/kg)	4.44 ± 0.92	3.94 ± 0.80 *	−0.50 ± 0.71	4.66 ± 0.77	4.44 ± 0.86	−0.22 ± 1.00	0.344
Exercise								
	HR (/min)	177.22 ± 14.28	181.44 ± 7.90	4.22 ± 9.72	186.00 ± 8.40	191.00 ± 9.57	5.00 ± 8.68	0.802
	HR (% age-predicted maximum HR)	89.33 ± 7.15	91.46 ± 4.03	2.13 ± 4.89	93.34 ± 4.45	95.86 ± 5.14	2.51 ± 4.34	0.806
	VT_1_ (mL/min/kg)	17.22 ± 4.60	16.06 ± 3.19	−1.17 ± 3.29	19.89 ± 3.20	18.06 ± 3.62	−1.83 ± 3.91	0.584
	VT_2_ (mL/min/kg)	25.56 ± 6.54	22.17 ± 4.72 *	−3.39 ± 4.79	27.78 ± 4.62	26.72 ± 5.69 **	−1.06 ± 4.39	0.137
	VO_2_peak (L/min)	2.18 ± 0.48	2.14 ± 0.49	−0.04 ± 0.24	2.42 ± 0.62	2.43 ± 0.63 **	0.01 ± 0.28	0.323
	BF (/min)	45.94 ± 9.25	54.44 ± 10.19 *	8.50 ± 9.30	48.11 ± 8.87	62.22 ± 12.75 *	14.11 ± 12.96	0.145
	RER	1.06 ± 0.06	1.29 ± 0.05 *	0.23 ± 0.08	1.07 ± 0.05	1.29 ± 1.28 *	0.21 ± 0.08	0.625
	SV (mL)	76.38 ± 17.04	74.20 ± 15.99	−2.17 ± 9.41	79.86 ± 18.31	78.62 ± 18.99	−1.24 ± 15.98	0.767
	CO (L/min)	13.47 ± 2.98	13.19 ± 3.02	−0.28 ± 1.52	14.92 ± 3.85	14.50 ± 3.49	−0.42 ± 2.83	0.481

BF, breathing frequency; CO, cardiac output; HR, heart rate; RER, respiratory exchange ratio; SV, stroke volume; VO_2_, oxygen consumption; VT_1_, first ventilator threshold; VT_2_, second ventilator threshold; VO_2_peak, peak oxygen consumption; *: *p* < 0.05 vs. before intervention, **: *p* < 0.05 vs. control group.

## Data Availability

The data are available on request to the corresponding author.

## Abbreviations

The following abbreviations are used in this manuscript:

BB	Biceps muscle
BF	Body fat
BMI	Body mass index
BMR	Basal metabolic rate
BW	Body weight
DBP	Diastolic blood pressure
ES	Effect size
FFM	Fat-free mass
FMs	Fat mass
FM	Femur
HM	Humerus
HIIT	High-intensity intermittent training
HR	Heart rate
HRmax	Maximal heart rate
MAP	Mean arterial pressure
MM	Muscle mass
NEAT	Non-exercise activity thermogenesis
REP	Rating of perceived exertion
RER	Respiratory exchange ratio
RF	Rectus femoris muscle
RR	Respiratory rate
SBP	Systolic blood pressure
SpO_2_	Partial oxygen saturation
TC	Triceps muscle
VCO_2_	Carbon dioxide production
VI	Vastus intermedius muscle
VO_2_	Oxygen uptake
VO_2_max	Maximal oxygen consumption
VT	Ventilatory threshold
WHO	World Health Organization
WHR	Waist–hip ratio
